# Exploration of heat and momentum transfer in turbulent mode during the precooling process of fruit

**DOI:** 10.1002/fsn3.1682

**Published:** 2020-07-02

**Authors:** Habibeh Nalbandi, Sadegh Seiiedlou

**Affiliations:** ^1^ Department of Biosystem Engineering Faculty of Agriculture University of Tabriz Tabriz Iran

**Keywords:** apple, designing, mathematical model, package, simulation

## Abstract

Developing a simulator is a prevalent method for the study of any process in which various phenomena occur simultaneously, such as the precooling process; it is also necessary in package designing. During the precooling process of fruit and in the case of large packages at high airflow rates, the flow regime inside the packages is turbulent, which is in most studies assumed to be a laminar flow that causes low prediction accuracy. In the present study, a mathematical model consisting of heat and momentum transfer in the case of a transient and a k‐ɛ turbulence model, respectively, was developed in the precooling process of fruits. Two packages and two airflow rates were used to validate the model. The results demonstrate that the turbulence‐model‐based simulation of the precooling was carried out with a lower element number, within a shorter time, and with a satisfactory accuracy (*R* ^2^ > .93866 & RMSE < 0.62). The model could predict the air movement between the fruit and consequently the heat transfer between the air and fruit. The simulator could be utilized to package designing and predicting the precooling time at the industrial scale to prevent the over‐cooling of fruits and reduce energy consumption. Based on the results, the precooling of apples in the commercial package was conducted in both experimental and simulation methods with high heterogeneity lasting 268 and 520 min at airflow rates of 0.5 and 1.5 L s^−1^ kg^−1^_p_, respectively. By using the developed simulator, the new package was designed for apple through which the cooling time and heterogeneity decreased 48% and 35%, respectively, as compared with those obtained in the commercial package.

## Graphical abstract

A mathematical model of a transient heat transfer and a k‐ɛ turbulence model for momentum transfer were utilized to develop a simulator for the precooling process of fruits. A new package was designed using the simulator to optimize the cooling time and heterogeneity.

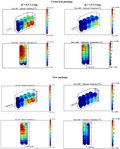

## 1 INTRODUCTION

Apple is one of the most valuable fruits in the world, being the second most consumed fruit globally. It is extremely rich in important antioxidants, flavonoids, and dietary fibers. The phytonutrients and antioxidants in apple may help reduce the risk of developing cancer, hypertension, diabetes, and heart disease. In 2014, the total world apple production was more than 84.63 million tons, 56.7% of which was in Asia. Iran, with 15.7 million tons in the same year, was ranked sixth in the world. Depending on their varieties, most apples are harvested and marketed from August to October. If regular cold storage is used, the marketing season can be extended through March, which is a very important time in Iran due to the new‐year holidays. Over the past decade, apple exports increased in Iran; however, the percentage is still low.

Apple needs to be delivered to consumers at its optimal quality after storage, transport, and distribution. It is a perishable fruit due to its high moisture content, ethylene production, susceptibility to microbial decay, and water loss (Anderson, Sarkar, Thompson, & Singh, 2004; Manganaris, Iliasb, Vasilakakisa, & Mignanic, 2007). It is, therefore, essential to use postharvest methods and technologies such as precooling process to reduce its loss and maintain its quality. In this process, cold air is forced through the fruit packed inside the packages located in the trays and pallets (Anderson et al., 2004; Becker, Misra, & Fricke, 1996; Brosnan & Sun, 2001; Castro, Vigneault, & Cortez, 2005; Chakraverty & Paul, 2001; Kader, 2002; Tutar, Erdogdu, & Toka, 2009).

Poor airflow distribution at different locations in fruit packages leads to differences in the airflow properties such as temperature, velocity, and turbulence (Alvarez & Flick, 1999a, 1999b; Amara, Laguerre, & Flick, 2004). Thus, each product receives different airflows depending on their position in the package, which can lead to the creation of considerable heterogeneities in the final temperature of products. The shape of products, spacing, arrangement, shape, and geometry of the package, package vent design, and total opening area are the parameters that affect the airflow distribution inside a package (Alvarez & Flick, 1999a, 1999b; Amara et al., 2004; Nalbandi, Seiiedlou, Ghassemzadeh, & Ranjbar, 2016). Among these factors, the vent position has a strong role in the airflow pattern. Based on findings of previous studies, vent area should be large enough not to restrict the airflow and to be well‐distributed on the package walls; also, they should not have a negative effect on the package structure (Castro et al., 2005; Ferrua & Singh, 2009; Tutar et al., 2009; Vigneault, Goyette, & Castro, 2006). In Iran, packages usually used for apple packaging do not have a good performance in the precooling process and storage, and some packages lead to high moisture loss of fruit during the storage and others to lower cooling rate. Thus, the vent position and its distribution on the package must be defined for an optimum and homogeneous precooling process.

Many researchers have used CFD modeling to design proper packages for fruits (Van der Sman, 2002; Zou, Linus, & McKibbin, 2006a, 2006b). Recent advances in computational resources and a decrease in the cost of modern computers have made the application of CFD modeling more efficient and popular, making it a powerful tool for simulation of the momentum transfer in most studies. Numerical modeling can reduce the costs of experiments and prototype equipment. Mathematical models capable of predicting the airflow pattern inside the package and fruit temperature variations have been used as ideal approaches by many researchers. In most of studies, researchers have assumed a laminar flow regime inside the packages (Van der Sman, 2002). Although the Re number is in the range of laminar flow in case of small packages, it increases in big packages due to a high airflow rate and a large hydraulic diameter. In the case of fruit packages, the flow is subjected to a sudden increase or decrease of the cross section repetitiously resulting in turbulence inside the packages. The heat transfer coefficient in the turbulent flow is higher than the laminar one, which leads to an increase in the rate of heat transfer between the product and cold air, and a decrease in the cooling time. Also, creating a turbulent flow inside the package may affect the cooling uniformity of the product. Besides, considerable turbulence is created when the air flows through the small vents. Therefore, the laminar inflow boundary condition could not be used in these zones. Despite these problems, the laminar flow regime assumption is usable, and the Navier–Stokes equations can also be used for turbulent flow simulations; however, it requires a large number of elements to capture all flow dynamics and solve the problem. High number of elements, long processing times, and a high RAM of a computer lead to an increase in the processing cost. An alternative is to consider the averaged equations, Reynolds‐averaged Navier–Stokes (RANS) equations, resulting in a hierarchy of equations and statistical unknowns. These unknown terms are modeled by equations known as closure relations, of which the eddy viscosity approach is the most common one.

The problems can be solved using lower element numbers via the turbulence model in a shorter time. A few studies were conducted base on the turbulent flow modeling of the precooling process. In some cases, the medium inside the packages was considered as a porous one, and the simulator was developed based on this assumption (Delele et al., 2016a, 2016b). This assumption leads to errors in the prediction, especially when the product to the package equivalent diameter ratio is lower than 10. O'Sullivan et al. (2016a, 2016b) developed a turbulent flow modeling for precooling of kiwi fruits packed in nonperforated poly liner bags. Thus, the air movement between the fruits was not simulated by the developed turbulence model, and the air transfer was only simulated between the packages and the pallet. Gruyters et al. (2019) studied the effect of box type, namely the corrugated cardboard “Mk4” box design and a reusable plastic crate (RPC), on the cooling time and uniformity by developing a CFD model. They found that the 7/8th cooling time of apple was 6.3 hr at a superficial air velocity of 0.258 ms^−1^ using the Mk4 box while it was 5.89 hr by the RPC box. According to their results, the cooling time, cooling uniformity, and energy consumption during the precooling of apple were lower by the application of RPC box than those obtained for the Mk4 box. However, the chilling injury was higher in the RPC box.

The aim of this research is to study the precooling process of apple by utilizing the turbulence airflow model for simulation of the precooling process of fruits, the airflow pattern, and the temperature field inside apple packages. This study also aimed at experimentally validating the developed model and assessing the sensitivity of apple cooling uniformity concerning the package vent designing in terms of the number of vents and distribution.

## 2 MATERIAL AND METHODS

### 2.1 Apple packages

**FIGURE 1:**
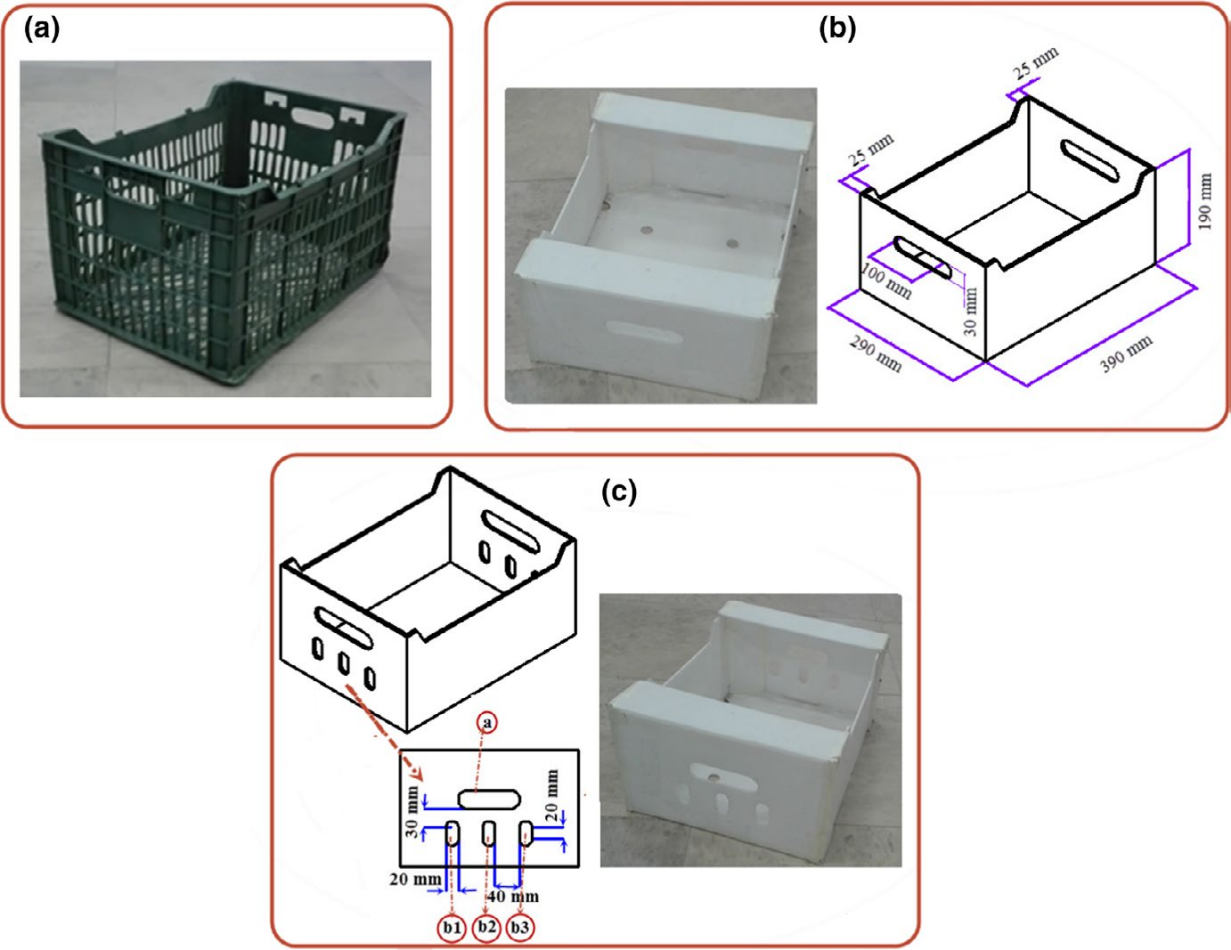
Various apple packages: (a) polyethylene package, (b) commercial package, and (c) newly designed package

In Iran, polyethylene packages are usually used for apple packaging, storage, and transportation. These packages have a lot of vents on all lateral walls and their bottoms (Figure 1a). Such a large open area leads to high loss of moisture in fruit during its storage in a cold room, even though fruit cooling is carried out quickly and uniformly. Besides, World Trade rules and standards do not allow using such packages. Hence, the use of corrugated plastic packages is generalized all over the world, including Iran. Figure 1b shows a commercial package for apple export with dimensions of 390 × 290 × 190 mm and a capacity of 7 kg packed in two rows. It has only one vent (vent “a” in Figure 1b) in the lateral walls for handling, and some small vents in the bottom of the box. Such a distribution of vents may not have a good performance in the precooling process. For optimum cooling and storage of apples, therefore, the vent position and its distribution must be defined on the package.

### 2.2 Simulation of the precooling process of apples

The turbulent flow is usually created during the precooling process of the fruit inside a large package and bin as well as a high airflow rate. The turbulence is created in the inlet or outlet vents due to their small area, resulting in a sudden increase in the air velocity. Inside the packages, repetitious changes in the cross section between the fruits and air direction lead to the creation of the turbulent flow. Therefore, the possibility of the turbulence model utilization for momentum transfer in the simulation of the precooling process of apples was studied and compared with the laminar model.

#### 2.2.1 Geometrical model and meshing

**FIGURE 2:**
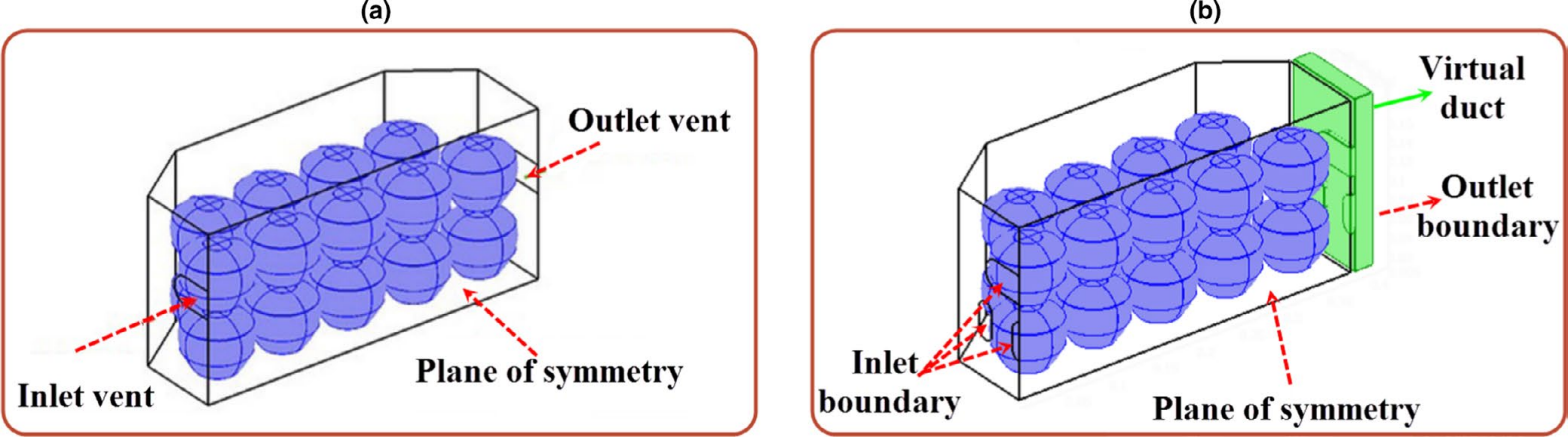
Geometrical model of the package: (a) commercial package and (b) new package

Creating a geometrical model of the problem was the first step for the simulation process. The geometrical model of a commercial apple package was created using the COMSOL MULTIPHYSICS software (Figure 2a). Only half of the package was modeled due to the symmetry condition. It consisted of 18 apples arranged in two layers without a tray. At the second step, the geometrical model was meshed using tetrahedral elements, along with the study of mesh independence. According to the results, 25,702 elements were enough to solve the problem with an acceptable accuracy (Figure S1 ). However, the final mesh consisting of 47,457 elements was selected to solve the problem with 24,992 elements in the air domain and 16,465 elements in the products domain.

#### 2.2.2 Mathematical models

In the following section, a mathematical model was used to simulate the precooling process of apples based on the turbulence model in case of momentum transfer and transient heat transfer in the product and fluid domain. The model was validated in commercial apple packages at different airflow rates. Then, its efficiency and usability were evaluated for other package designs.

##### Reynolds number

Due to the shape and arrangement of fruits, some pore spaces were created among the fruits in the packages, which resulted in the changes of air passing area. Such changes in the cross‐sectional area lead to a change of the Reynolds number (Re) due to changes of hydraulic diameter. To calculate Re number, therefore, two sections (Figure S2) were selected with maximum (a) and minimum (b) pore areas inside the package along the air passing direction. Re number was then calculated using Equation 1, where *D* is the hydraulic diameter, which in turn was calculated using Equation 2. Re number in the inlet vent was obtained in the same manner.

(1) $$\text{Re}=\frac{\rho_{a}UD}{\eta }$$

(2) $$D=\frac{4A}{\text{Pp}}$$

Considering that the usual airflow rate for forced‐air precooling of apples was in the range of 0.5–1.5 L s^−1^ kg^−1^_p_, Re number was also calculated at minimum and maximum flow rates. At an airflow rate of 0.5 L s^−1^ kg^−1^_p_, Re numbers were 570, 725, and 4,400 at two cross sections and inlet vent, respectively. The calculated numbers were 1,710, 1,275, and 13,200 at an airflow rate of 1.5 L s^−1^ kg^−1^_p_ at the same area. The flow regime was turbulent in the vents of both airflow rates and laminar inside the packages and at both the cross‐section and airflow rate. However, it was considered as a turbulent flow due to the changes in the air direction.

##### Momentum equation‐Turbulence model

Reynolds‐averaged Navier–Stokes (RANS) equation was selected to simulate the momentum transfer during the precooling process of apples. It includes two main models, namely k‐ε and k‐ω. The k‐ε model is one of the most used turbulence models for industrial applications. It assumes that the flow is incompressible and that the fluid is Newtonian. The equations for the momentum transport and continuity are Equations 3 and 4:

(3) ∇.u=0

(4) $$\rho_{a}\frac{\partial u}{\partial t}+\rho_{a}\left(u.\nabla \right)u=\nabla \left[-\text{PI}+\left(\eta +\eta_{T}\right)\left(\nabla u+{\left(\nabla u\right)}^{T}\right)\right]$$

where *η*_T_ denotes the turbulent viscosity modeled by Equation 5. The k‐ε model introduces two additional transport equations and two dependent variables: the turbulent kinetic energy, *k*, and the dissipation rate of turbulence energy, *ε*, which are shown in Equations 6 and 7, respectively.

(5) $$\eta_{T}=\rho_{a}C_{\mu }k_{T}^{2}/\varepsilon$$

(6) $$\rho_{a}\frac{\partial k_{T}}{\partial t}+\rho_{a}u.\nabla k_{T}=\nabla \left[\left(\eta +\eta_{T}/\sigma_{k}\right)\nabla k_{T}\right]+\eta_{T}P\left(u\right)-\rho_{a}^{\varepsilon }$$

(7) $$\rho_{a}\frac{\partial \varepsilon }{\partial t}+\rho_{a}u.\nabla \varepsilon =\nabla \left[\left(\eta +\eta_{T}/\sigma_{\varepsilon }\right)\nabla \varepsilon \right]+C_{\varepsilon 1}\varepsilon \eta_{T}P\left(u\right)/k_{T}-C_{\varepsilon 2}\rho_{a}\varepsilon^{2}/k_{T}$$

(8) $$P\left(u\right)=\nabla u\left(\nabla u+{\left(\nabla u\right)}^{T}\right)$$

The model constants in the above equations are *C_µ_* = 0.09, *C_ɛ_*_1_ = 1.44, *C_ɛ_*_2_ = 1.92, *σ_k_* = 1, and *σ_ɛ_* = 1.3. Turbulent isotropic and isotropic diffusion (tuning parameter) diffusions were assumed as 0.25 and 0.5, respectively. The air properties were selected as the same dry air, and airflow rates were considered as 1.5 and 0.5 L s^−1^ kg^−1^_p_. The following boundary condition was used to complete the airflow model. The boundary conditions used to complete the airflow model were described in Table 1.

**TABLE 1:** Boundary conditions for the airflow model equations

Inlet: Pressure no viscous stress	$\begin{array}{cc} p=p_{0}, & \left(\eta +\eta_{T}\right)\left(\nabla u+{\left(\nabla u\right)}^{T}\right)n=0\\ k=k_{0}, & \varepsilon =\varepsilon_{0} \end{array}$
Wall: logarithmic wall function	$\begin{array}{cc} n.u=0 & \left(\eta +\eta_{T}\right)\left(\nabla u+{\left(\nabla u\right)}^{T}\right)n=\left[\rho_{a}C_{\eta }^{0.25}k^{0.5}/\left(\ln\left(\partial_{W}^{+}\right)/k+C^{+}\right)\right] \end{array}u$ $\begin{array}{cc} n.\nabla k=0, & \varepsilon =C_{\eta }^{0.75}k^{1.5}/ \end{array}\left(k\partial_{W}\right)$ $\partial_{W}^{+}=\partial_{W}\rho_{a}C_{\eta }^{0.25}k^{0.5}/\eta$
Outlet: Velocity (outlet velocity is uniform and normal to the outlet section)	$\begin{array}{c} u=u_{0}\\ \begin{array}{cc} n\nabla k=0, & n.\nabla \varepsilon =0 \end{array} \end{array}$
Symmetry boundary	$\begin{array}{c} \begin{array}{cc} n.u=0, & t.\left[-\text{PI}+\left(\eta +\eta_{T}\right)\left(\nabla u+{\left(\nabla u\right)}^{T}\right)\right]n= \end{array}0\\ n.\left[\left(\eta +\eta_{T}/\sigma_{k}\right)\nabla k=\rho_{a}uk\right]=0\\ n.\left[\left(\eta +\eta_{T}/\sigma_{\varepsilon }\right)\nabla \varepsilon =\rho_{a}u\varepsilon \right]=0 \end{array}$

##### Heat transfer model for fluid and product domain

The heat transfer equation within the fluid domain is written as Equation 9, which describes a time‐dependent process that includes the conduction and convection terms.

(9) $$\rho_{a}{\text{Cp}}_{a}\frac{\partial T_{a}}{\partial t}+\rho_{a}{\text{Cp}}_{a}\left(u.\nabla T_{a}\right)=\nabla .\left(k_{a}\nabla T_{a}\right)$$

Transient heat transfer in the product domain was as follows (Equation 10):

(10) $$\rho_{p}Cp_{p}\frac{\partial T_{p}}{\partial t}=\nabla .\left(k_{p}\nabla T_{p}\right)$$

The appropriate boundary conditions for these equations are summarized in Table 2.

**TABLE 2:** Boundary conditions for heat transfer equations in the fluid and product domain

Fluid domain	
Boundary conditions	Inlet: *T*_a_ = *T*_a0_ Outlet: $\left(-k_{a}\nabla T_{a}\right)n=0$ Wall: $\left(k_{a}\nabla T_{a}\right)n=0$ (The wall are perfectly insulated) Interface: *T*_a_ = *T*_P_ Symmetry plane: $\left(k_{a}\nabla T_{a}\right)n=0$
Air properties	Similar to those of dry air at 1°C
Product domain	
Boundary conditions	$\left(k_{P}\nabla T_{P}-k_{a}\nabla T_{a}\right)n=0$
Apple properties	*k*_p_ = 0.5 w/m.°C *C*_p,p_ = 4.241 kJ/kg.°C *ρ*_p_ = 800 kg/m^3^
Initial temperature	21°C
Assumption	No moisture loss during the cooling

#### 2.2.3 Solving methods

The mathematical models were solved using the COMSOL MULTIPHYSICS software (version 3.5), and PARDISO was selected as the linear system solver. It took about 49 min to solve the coupled heat and momentum transfer equations using a personal computer with 32 GB RAM (Processor: Intel^®^ Core™ i7‐2700K CPU@ 3.50 GHz, 3.50 GHz).

### 2.3 Laminar model

Considering a laminar flow regime inside the package, the laminar flow was selected in this step to simulate the momentum transfer phenomena during the precooling process of apples. Equations 3 and 11 give the mass and momentum conservations for air in the system, respectively. The constant velocity and pressure were defined as the boundary condition at the outlet and inlet vents, respectively. No‐slip boundary condition was applied to all solid surfaces, and symmetry boundary condition was used on the plane of symmetry. Heat transfer equation was the same (Table 2). Also, the geometrical model, mesh element, and solving method were considered the same as the previous ones.

(11) $$\rho_{a}\frac{\partial u}{\partial t}+\rho_{a}\left(u.\nabla u\right)=-\nabla P+\nabla .\left[\mu_{a}\left(\nabla u+{\left(\nabla u\right)}^{T}\right)\right]$$

### 2.4 Experimental study

**FIGURE 3:**
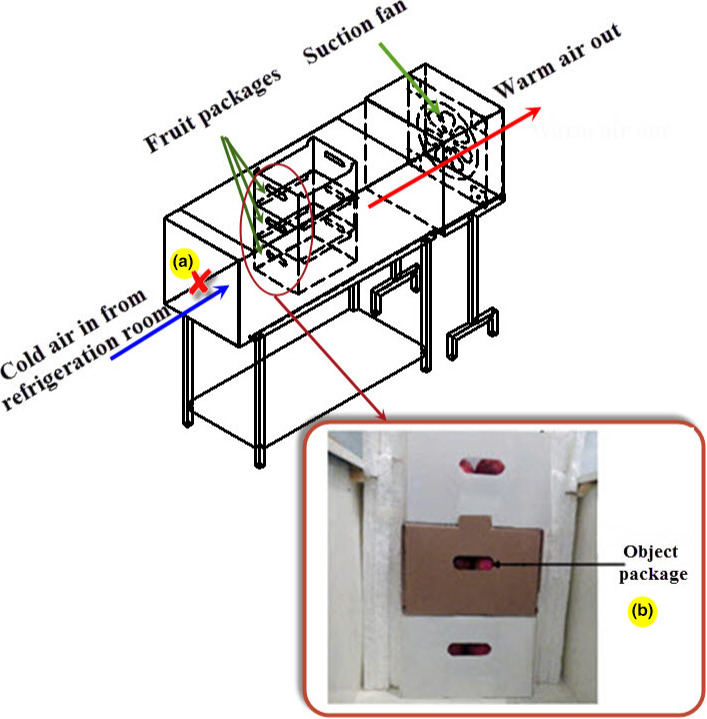
Horizontal wind tunnel: (a) position of airflow rate instrument (center of cross section); (b) position of thermocouples

**FIGURE 4:**
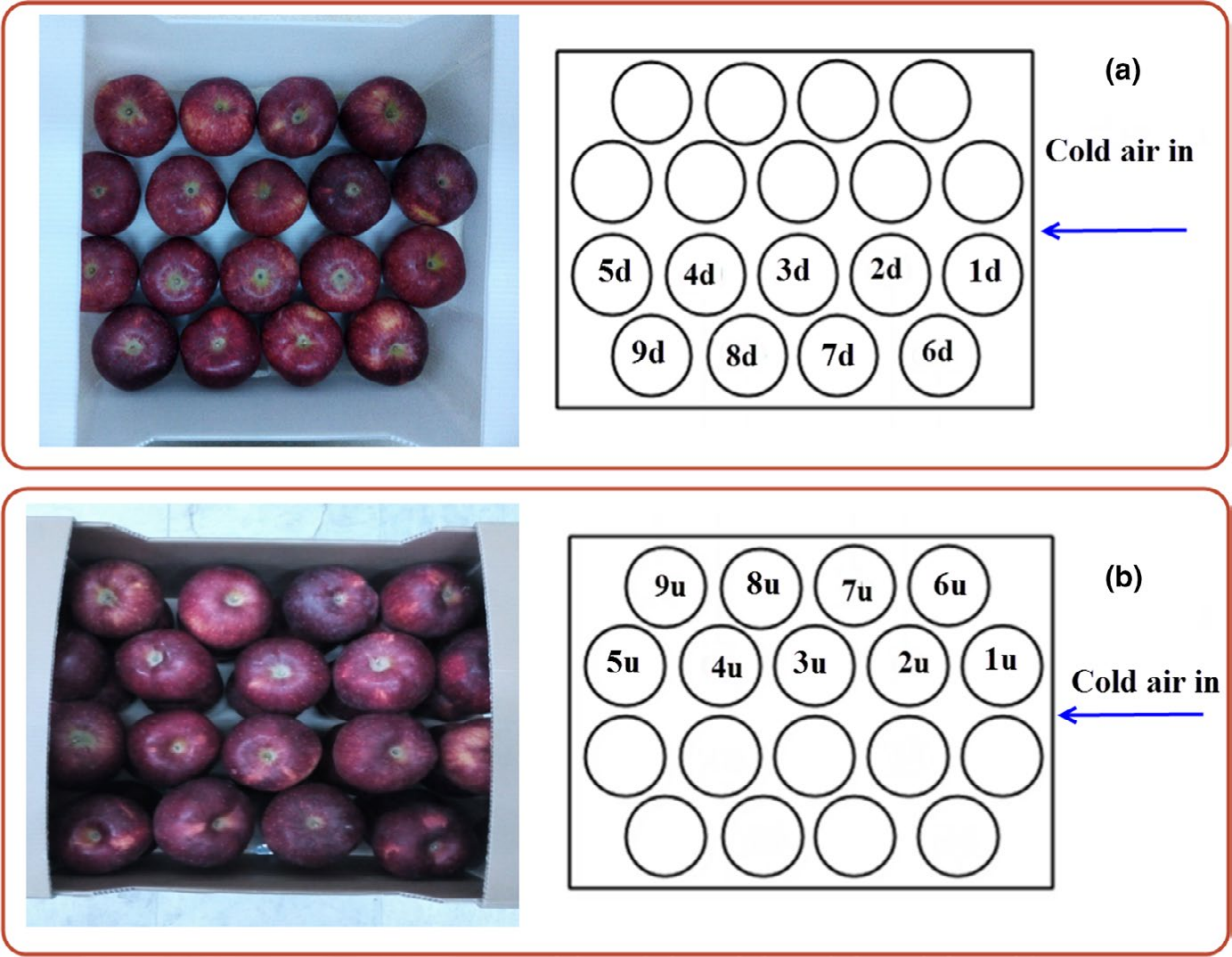
Instrumented apple location inside the commercial package (top view): (a) down row (No 2d, 4d, 6d, and 8d) and (b) up row (No 2u, 4u, 6u, and 8u)

To validate the model, an experimental study was performed using the forced‐air precooling system. It consisted of a suction fan to generate a desirable airflow rate and a horizontal tunnel to place the fruit packages. The cold air from the cold storage entered the system and exited the tunnel after cooling the fruits (Figure 3). Three packages were placed inside the center of the tunnel. Eight pieces of fruit were instrumented via K‐thermocouple wire to measure the fruit temperature variations during the process (Figure 4), which were used for the model validation. An acquisition system, including a PC and a data logger, was used to collect data. The temperature was saved every 1 min. The exact temperature of airflow entering the system was recorded by a thermocouple located in the front of the tunnel. The average temperature of the air was 0.3°C. The experiments were carried out at two airflow rates of 0.5 and 1.5 L s^−1^ kg^−1^_p_, and an air temperature and humidity of 1°C and 80%, respectively. The process was continued up to 7/8th temperature of the fruit.

### 2.5 New package design

After the experimental validation, the best model was selected to simulate the precooling process of fruits in the case of big packages and bins. In this section, another design of package was simulated, and the obtained results were validated experimentally for two reasons. Firstly, it was intended to evaluate the developed model in the case of other packages. Therefore, a commercial apple package was modified in terms of designing and distribution of vents. Secondly, the aim was to improve the cooling process of apples, such as decreasing the cooling time and heterogeneity.

According to the results of the simulation in the previous section, the cooling process of apples inside the commercial package was not uniform, and the cooling of fruits located in the bottom row was slower than those in the top one. Therefore, extra vents consisting of three vents (b1, b2, and b3 in Figure 1c) were designed in the bottom of old vents (“a” in Figure 1b) on the lateral walls of the commercial package to improve the cooling uniformity. In the new package, the air distribution was not clear between the vents; therefore, a virtual duct was considered in the outlet side of the package (Figure 2b). This virtual duct allowed the simulator to find the exact air distribution in the outlet vents. The outlet boundary condition was described in the outer side of the virtual duct. Then, the simulation process was carried out using the same method as described previously, along with the study of mesh independence. According to the results, 25,702 elements were enough to solve the problem with acceptable accuracy. The simulation time was about 243 min. Besides, this new package was studied experimentally, followed by validating the simulation results.

## 3 RESULTS AND DISCUSSION

### 3.1 Evaluation of the turbulence and laminar model

**FIGURE 5:**
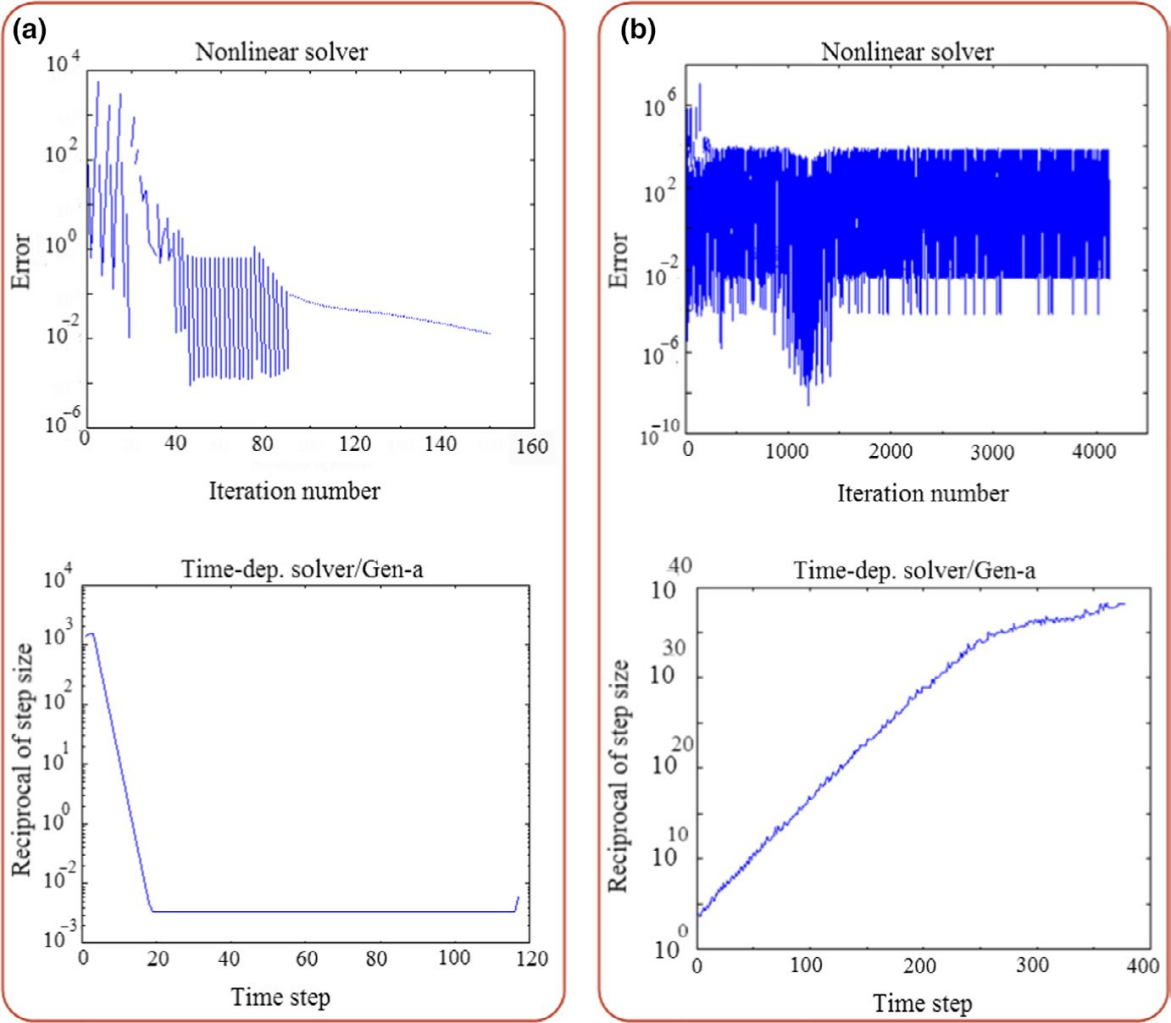
Solving error versus iteration number and reciprocal of step size versus time step (a) turbulence model, and (b) laminar model

The mathematical model based on the transient heat transfer in the fluid and product domain, as well as the k‐ε turbulence model in the momentum transfer, was used to simulate the cooling process of apples inside the commercial package. It took about 49 min to solve the equations simultaneously. The graph of solving error versus the iteration number, and the reciprocal of step size versus time step is shown in Figure 5a. The reciprocal of step size was constant, the solving error gradually decreased, and the numerical solution was converged after about 20 time steps. As mentioned in the previous section, the momentum transfer was also modeled based on the laminar flow inside the package. As it is clear in Figure 5b, the numerical solution of laminar model equations is not converged at the same condition of the model developed based on the turbulent flow, and it needs high mesh density. Therefore, using the turbulence model for momentum transfer could simulate the precooling process with lower elements resulting in a shorter simulating time. This is a very important point for the developed simulator to make it usable in industrial applications. Therefore, the simulator validated for real‐time controlling of the industrial precooling process can be utilized to prevent over‐cooling of fruits and to reduce energy consumption.

### 3.2 Experimental validation of the selected model

**FIGURE 6:**
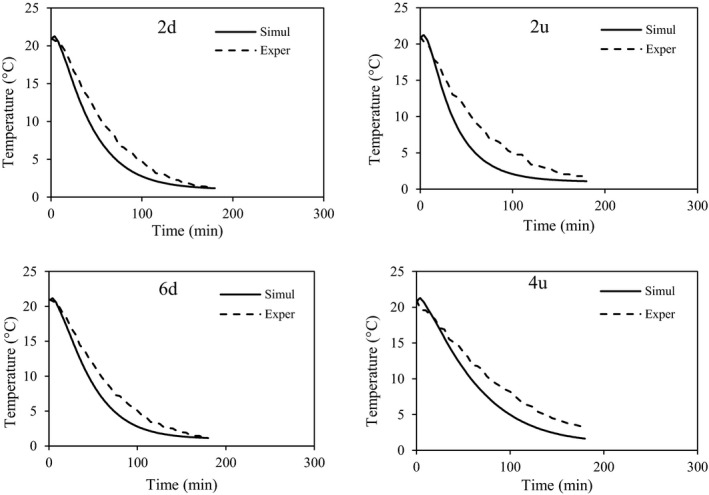
Experimental and simulated temperatures of some fruits inside the commercial package at an airflow rate of 1.5 L s^−1^ kg^−1^_p_

**FIGURE 7:**
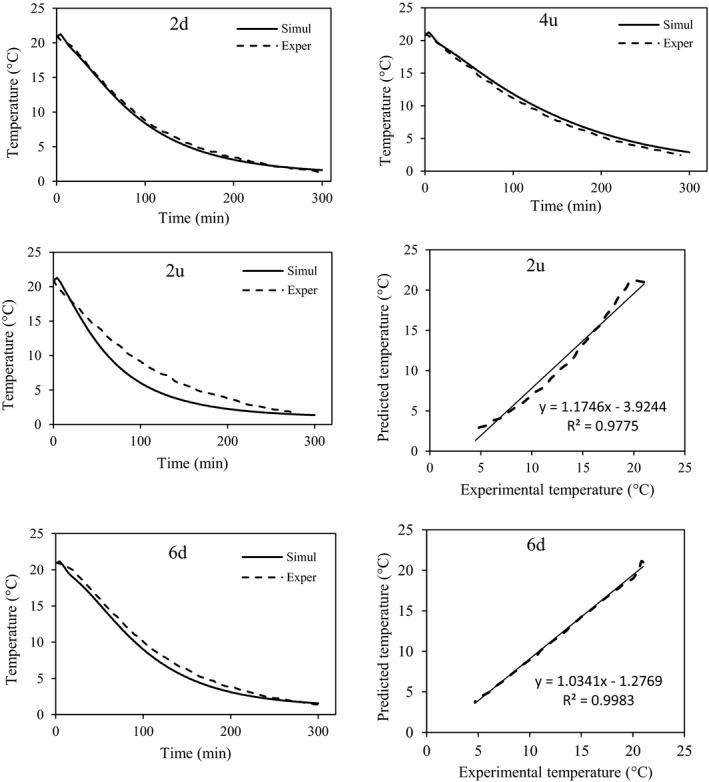
Experimental and simulated temperatures of some fruits inside the commercial package at an airflow rate of 0.5 L s^−1^ kg^−1^_p_

To validate the selected model (the k‐ε turbulence model) and to measure the central temperature of some fruits, an experimental study was performed at two airflow rates (Figure 4). Figures 6 and 7 show the experimental and simulated temperatures of the fruits at airflow rates of 1.5 and 0.5 L s^−1^ kg^−1^_p_, respectively. Differences between the experimental and predicted data were in the ranges of 0.1 to 1.37°C and 0.15 to 0.45°C at airflow rates of 1.5 and 0.5 L s^−1^ kg^−1^_p_, respectively. Some statistical indexes, such as *R* ^2^ and RMSE, were used to confirm the prediction accuracy of the model. At an airflow rate of 1.5 L s^−1^ kg^−1^_p_, *R* ^2^ and RMSE were in the range of .93866–.99747 and 0.10720–0.45569, respectively (Table 3). The highest prediction error was related to the point of 2u (top row) that could be due to the experimental errors such as thermocouple reading errors or its moving from a specific location; at the other points, however, the prediction accuracy was acceptable. At an airflow rate of 0.5 L s^−1^ kg^−1^_p_, the model also had a good prediction of fruit temperatures at most of the points; at point 9u, however, the prediction did not show high accuracy. In Figure 7, the experimental and predicted temperatures are shown as a linear regression. This line slope indicates the prediction error. For example, the line slopes were 1.1746 and 1.0341 for points 2u and 6d, respectively. A high value of the line slope shows a high prediction error. Considering that the values of prediction errors are not the same at all the studied points, it can be concluded that the experimental error may be the main reason for this situation (Table 3). The model assumption, such as no moisture loss, could affect the model accuracy. However, it is the same at all points and conditions.

**TABLE 3:** Mathematical model of momentum transfer base on the laminar flow and boundary conditions

Incompressible fluid and laminar flow	∇.u=0 $\rho_{a}\frac{\partial u}{\partial t}+\rho_{a}\left(u.\nabla u\right)=-\nabla P+\nabla .\left[\mu_{a}\left(\nabla u+{\left(\nabla u\right)}^{T}\right)\right]$
Boundary conditions	Inlet: p = p_0_ Wall: **u** = 0 Outlet: **u** = **u_0_** Interface: **u** = 0

A similar condition was reported by Zou et al. (2006a), Zou et al. (2006b) who found about 2 K differences after 4 hr of cooling between their developed model prediction and experimental data, with about 3–4 K differences in some points. Inaccurate positioning of thermocouples and model input and assumption were the main reasons for errors. In a model developed by Ferrua and Singh (2009), the precooling of strawberry also had a satisfactory prediction whereby 0.7°C differences were observed between experimental and predicted average fruit temperatures per package. Average estimated errors for strawberry temperatures were 1 and 0.8°C in two different packages, respectively, obtained by Nalbandi et al. (2016).

As a conclusion, the developed model had an acceptable accuracy for simulating the precooling process of fruits in the case of big packages at various airflow rates. At both rates, however, the developed model underestimated the error in predicting the fruit temperatures. In the present study, *k*_0_ and *ɛ*_0_ values in the case of fruit packages were considered as 0.005 m^2^s^−2^ and 0.005 m^2^s^−3^, respectively, due to the lack of information about the two parameters. To improve the possibility of model accuracy, future researchers are recommended that to study the effect of this parameter on the model accuracy and conduct thermal sensitivity analysis.

### 3.3 Application of the results of the selected model to evaluate the precooling process of apples in the commercial package

**FIGURE 8:**
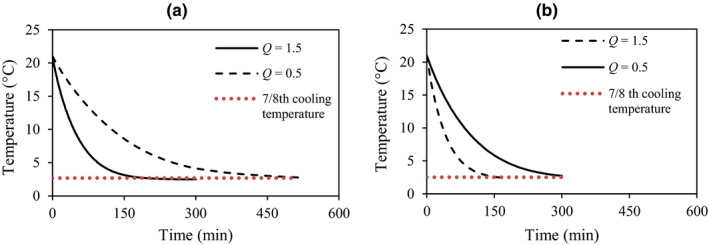
Average fruit temperature versus cooling time; (a) commercial package and (b) new package

**FIGURE 9:**
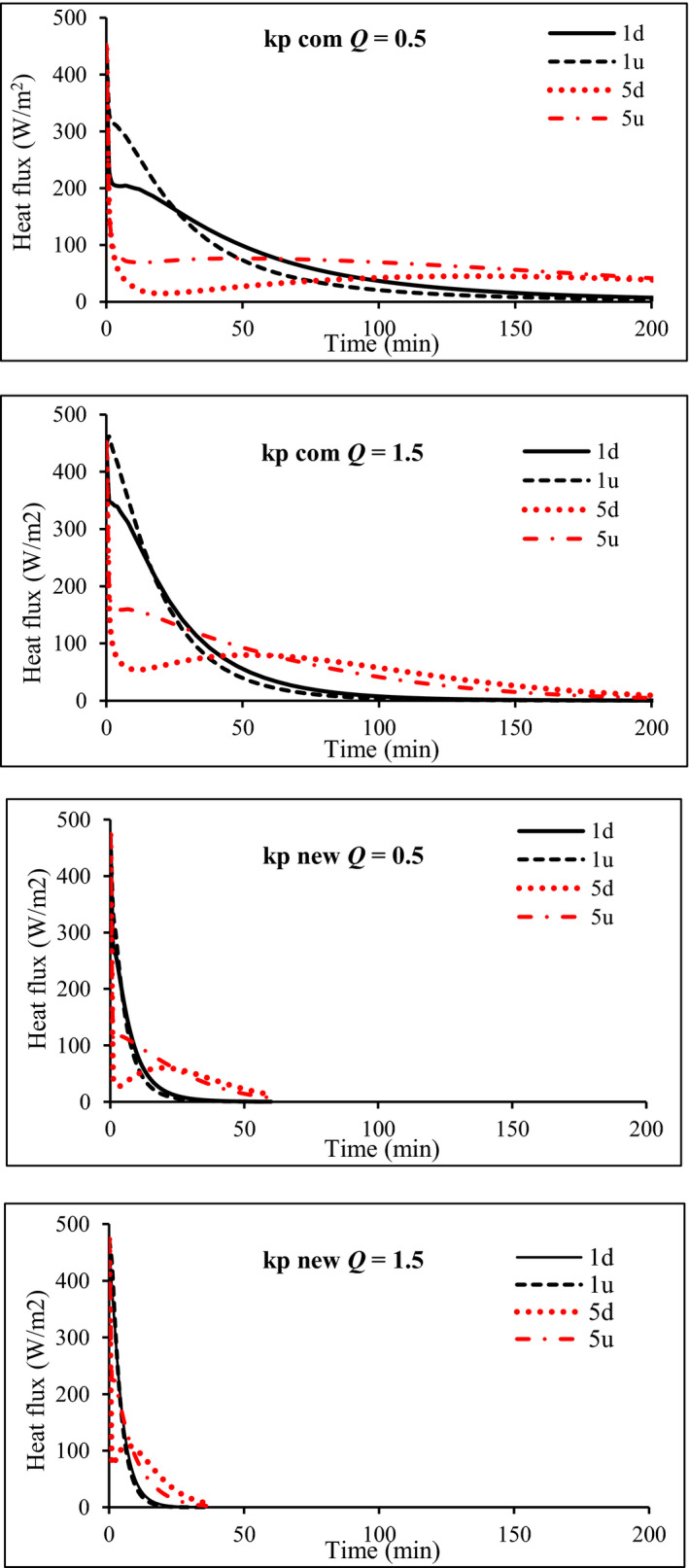
Conduction heat flux of some fruits versus cooling time

The developed simulator was validated using a commercial package of apples. Therefore, the precooling process of apples was also studied here. Based on the validation data, the volume average predicted 7/8th cooling times of apples were 268 and about 520 min at airflow rates of 1.5 and 0.5 L s^−1^ kg^−1^_p_, respectively. Figure 8 shows the average fruit temperatures versus cooling times. As it was assumed, the cooling of fruits occurred faster at the high airflow rate in a way that increasing the airflow rate from 0.5 to 1.5 L s^−1^ kg^−1^_p_ reduced the cooling time to about 48 percent. In simple terms, the higher airflow rate had higher cooling rates. The conduction heat flux of some fruits is shown in Figure 9. The fruits located at the bottom layer had lower heat flux than those located at the top row. At an airflow rate of 1.5 L s^−1^ kg^−1^_p_, conductive heat flux had a higher rate than that of 0.5 L s^−1^ kg^−1^_p_. The average conductive heat fluxes for fruits 1d were 27.5 and 35.7 Wm^−2^.min, respectively. The same condition was observed for other fruits.

Similar results were also reported by other researchers (Castro, Vigneault, & Cortez, 2004; Castro et al., 2005; Cortbaoui et al., 2006; Vigneault et al., 2006). However, Kumar, Kumar, and Murthy (2008) found that there were critical air velocities above which airflow rates had no significant effect on the cooling rates of products. For instance, the rates were 3.5 and 2.6 ms^−1^ for oranges and tomatoes, respectively.

**FIGURE 10:**
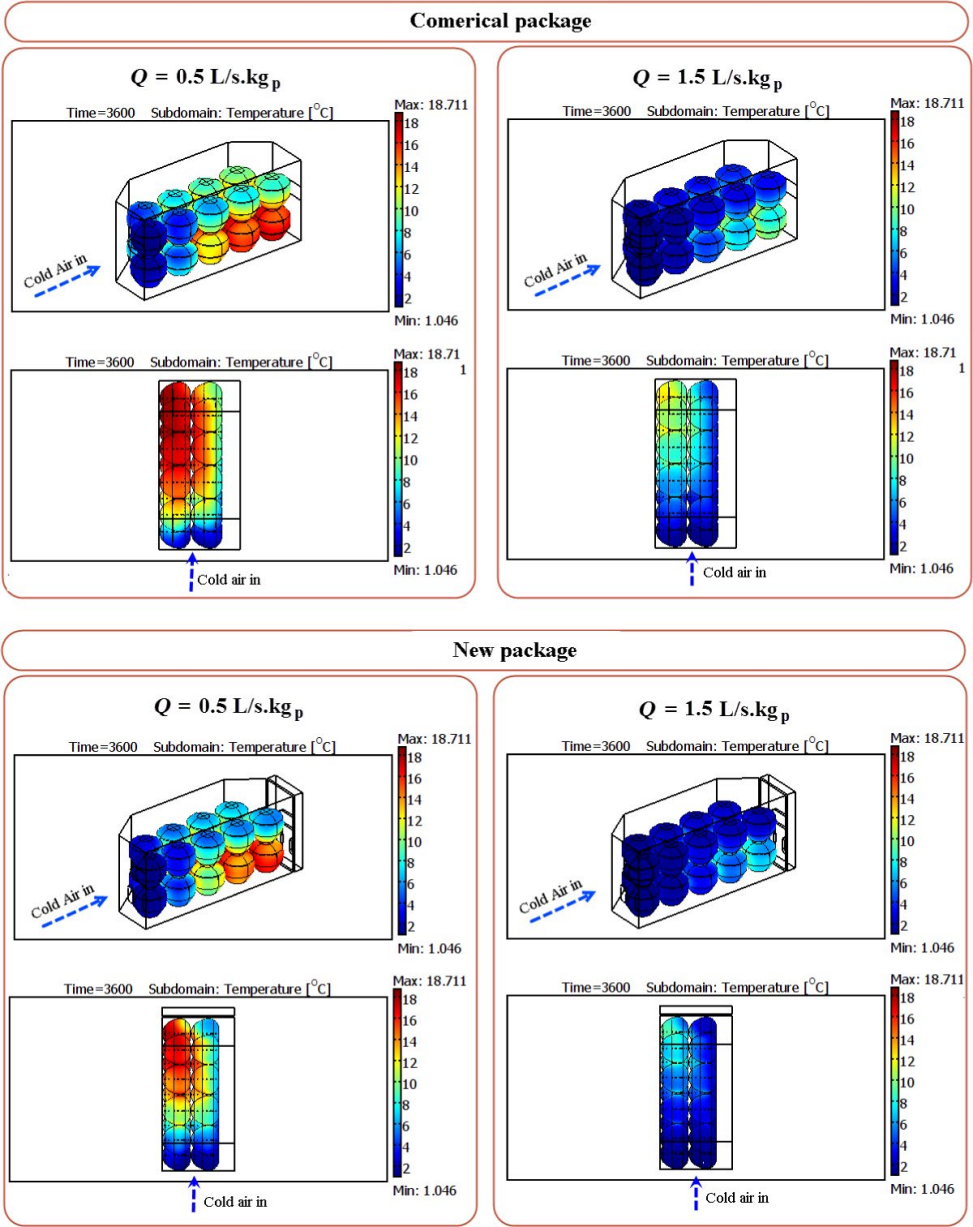
The surface temperature of apples within the commercial and new packages at the studied airflow rates

**FIGURE 11:**
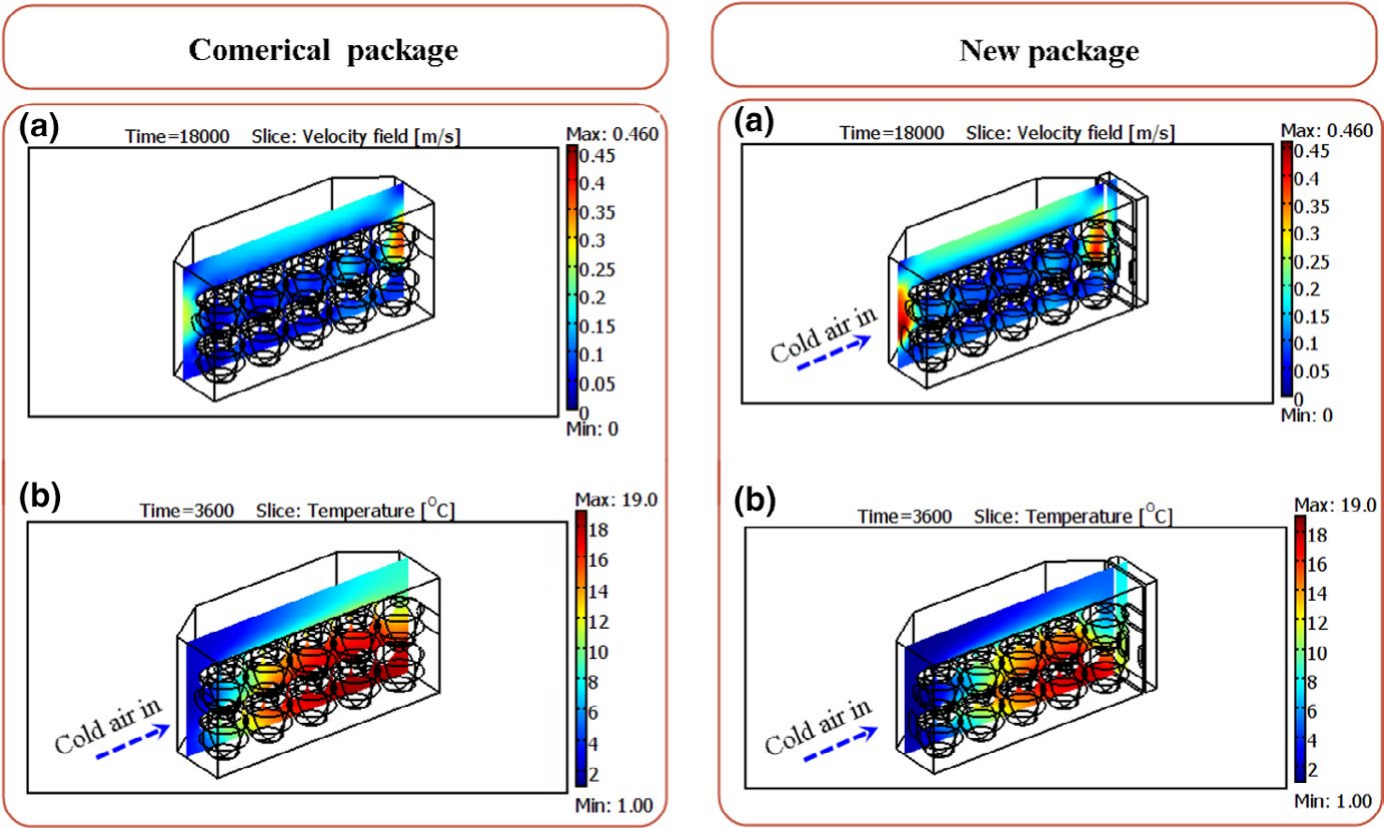
Profile of air velocity and temperature within the commercial package at an airflow rate of 0.5 L s^−1^ kg^−1^_p_

According to the results, the cooling process of apple at both airflow rates was performed with high heterogeneity in the commercial package. At an airflow rate of 0.5 L s^−1^ kg^−1^_p_, there were about 270 min differences between the 7/8th cooling time of fruits. As shown in Figure 10, the cooling rate of the fruits located in the bottom layer was lower than those in the top one. Due to such heterogeneity, some fruits overcooled and the others did not cool enough and did not reach the 7/8th cooling temperature. The air velocity distribution inside the packages (Figure 11a) indicated that a large fraction of cold air flew through the top section of the package due to the low resistance, and the fruits located in this area were cooled faster than those in the bottom of the package. Also, the fruits located in the second part of the package were cooled slower than those in the first half. The convective heat transfer between cold air and the product led to an increase in the air temperature during traveling along the package and a decrease in the heat transfer rate (Figure 11b). Such heterogeneity is always created inside the packages where the in‐series cooling is the dominant method of fruit cooling (Ferrua & Singh, 2009; Nalbandi et al., 2016). The same manner was observed in an airflow rate of 1.5 L s^−1^ kg^−1^_p_ in which a difference of 170 min was observed between the 7/8th cooling times of fruits (Figure 10). The high airflow rate led to a lower rate of air temperature increase during its traveling along with the package.

These results indicate that the in‐series method of cooling inside the packages makes it impossible to omit the heterogeneity between the fruits located in the first and second parts of the package unless using a higher airflow rate. However, modifying the design of vents in the commercial packages could improve the cooling uniformity between the top and bottom layers of fruits so that the latter receives a higher fraction of airflow.

#### 3.3.1 Moisture loss

Moisture loss of apples was studied during the cooling process by measuring their initial and final masses. Fruits lost about 0.12% and 0.04% of their initial masses at airflow rates of 0.5 and 1.5 L s^−1^ kg^−1^_p_, respectively. The moisture loss was decreased in spite of increasing the airflow rate, is in appositive of previously results reported (Thompson, 2003). In most conditions, the high airflow rate led to higher moisture loss. Air velocity profile was lower than 0.05 ms^−1^ inside the package at the bottom layer of fruits and only those located at the top row were in contact with the cold air of high velocity. Therefore, the moisture loss of fruits in the bottom layer was affected lightly by an increase in the airflow rate because of their minimum contact with the cold air. Instead of increasing the moisture loss, therefore, increasing the airflow rate decreased the total moisture loss by decreasing the cooling time.

### 3.4 New package design

Any developed model should be validated at various conditions. The proposed model was validated at two airflow rates using the commercial package. It was also validated using another design of the packages. However, only the shape and number of vents were changed in the new packages.

**FIGURE 12:**
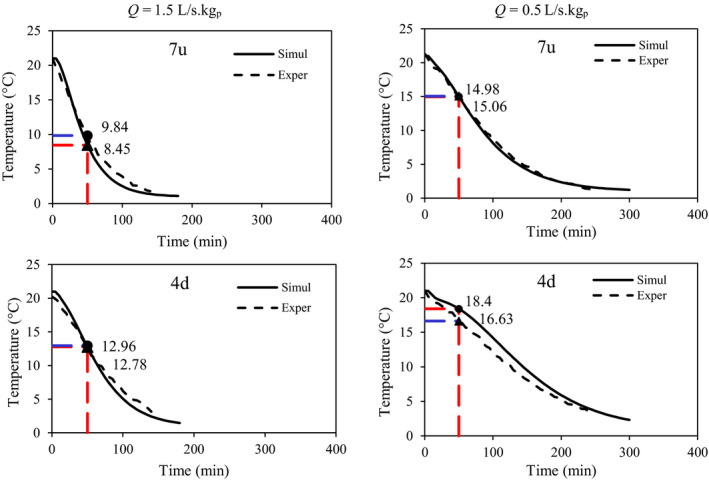
Experimental and simulated temperatures of some fruits inside the new package

The vents of the package were redesigned due to the decreases in the observed heterogeneity of the commercial package and the cooling time. Three extra vents (b1, b2, and b3 in Figure 1c) were designed in the lateral walls of the package, and the precooling process was simulated inside the new package. The predicted temperatures of some fruits are shown in Figure 12. The developed model was also validated experimentally in the new packages and a good agreement was observed in the data (Table 3). The results corroborate that the model can be used for simulation of fruit cooling at any airflow rate and various package designs. As it is clear from Figure 12, maximum and minimum differences between the predicted and measured temperatures were 1.77 and 0.08°C, respectively.

**FIGURE 13:**
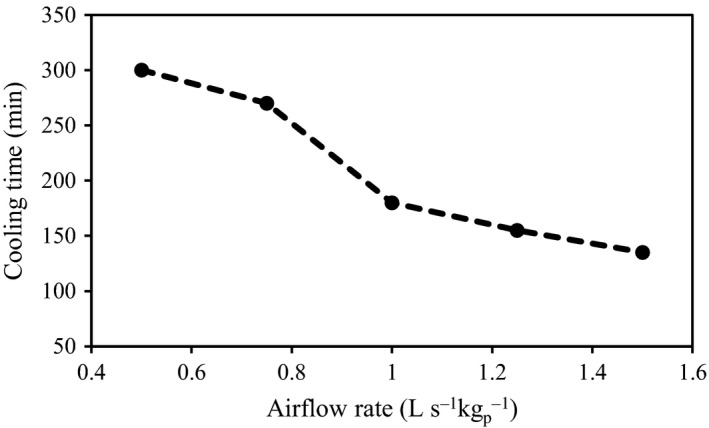
Sensitivity analysis based on the effect of airflow rate increment on the cooling time

The results of the simulation showed that the cooling process was carried out more rapidly (Figure 8b) and the average 7/8th cooling time (155 min) of the fruits declined as compared to that of the commercial package (268 min) at an airflow rate of 1.5 L s^−1^ kg^−1^_p_. The cooling time also decreased about 220 min in the new package at an airflow rate of 0.5 L s^−1^ kg^−1^_p_. Sensitivity analysis showed that a 76% increase in the vent area (the new package versus the commercial one) led to decreased cooling times of about 42% and 46% at airflow rates of 0.5 and 1.5 L s^−1^ kg^−1^_p_, respectively. In addition, sensitivity analysis was performed based on the airflow rate increment and its effect was studied on the cooling time. According to the results, an increment in the airflow rate (step size: 25%) led to a decrease in the cooling time. However, its effect was different at each step. The maximum decreasing in the cooling rate was observed when the airflow rate increased from 0.75 to 1 L s^−1^ kg^−1^_p_ so that the cooling time decreased about 33.3%. More increasing in the airflow rate had lower effect. For example when the airflow rate increased from 1 to 1.25 L s^−1^ kg^−1^_p_ and 1.25 to 1.5 L s^−1^ kg^−1^_p_ the cooling time decreased about 13% and 12% (Figure 13).

The cooling uniformity in the new package so that the maximum difference between the 7/8th cooling times of fruits was 110 min at an airflow rate of 1.5 L s^−1^ kg^−1^_p_ (Table 4). Although the fruits located in the second part of the package cooled more slowly, the cooling of those placed at the top and bottom layers was more uniform than the ones in the commercial package. The uniform distribution of cold air between the fruits is the main reason for cooling uniformity (Figure 11a). In the new package and an airflow rate of 0.5 L s^−1^ kg^−1^_p_, about 57% of airflow rate flowed through the top vent (vent a in Figure 1c, the previous vent in the commercial package) and 43% from the new vents (30% from vents b1 and b3 and 13% from vent b2). This airflow increased the cooling rate of fruits located in the bottom row. Therefore, increasing the number and area of vents led to decreased cooling time and increased cooling uniformity. This situation was the same at the two airflow rates.

**TABLE 4:** Prediction accuracy of the developed model at various conditions

Airflow rate (L s^−1^ kg^−1^_p_)	2d	2u	4d	4u	6d	9u
Commercial package						
1.5						
RMSE	0.30219	0.45569	0.15648	0.35780	0.3046	0.10720
*R* ^2^	.97050	.93866	.99747	.97827	.97253	.99630
0.5						
RMSE	0.07178	0.39777	0.42125	0.09235	0.15486	0.62516
*R* ^2^	.99869	.97753	.97343	.99881	.99831	.97846
New package						
1.5						
RMSE	0.263836	0.290953	0.17711	0.144487	0.354635	0.3118
*R* ^2^	.981335	.973614	.996166	.996344	.960338	.988157
0.5						
RMSE	0.183516	0.144051	0.197709	0.058042	0.27189	0.115814
*R* ^2^	.984634	.982546	.989064	.998846	.961786	.998177

Measuring the moisture loss during the precooling process of apples using the new package indicated that increasing the opening area had no significant effects on the moisture loss with values of 0.06% and 0.03% at airflow rates of 0.5 and 1.5 L s^−1^ kg^−1^_p_, respectively.

A comparison of the commercial and new package (Table 5) indicates that the new package is more suitable for apple precooling process due to a lower cooling time and high uniformity than the commercial one.

**TABLE 5:** Comparison of the commercial and new package

Evaluation index	Airflow rate (L s^−1^ kg^−1^_p_)	Commercial package	New package
7/8th cooling times (min)	1.5	268	155
	0.5	520	300
Maximum temperature difference (°C)	1.5	170	110
	0.5	270	200
Moisture loss (%)	1.5	0.04	0.03
	0.5	0.12	0.06

## 4 CONCLUSION

In the present study, a new mathematical model was developed to simulate the heat and momentum transfer during the precooling process inside large fruit packages and bins where a turbulent flow is created. The developed model was validated at two airflow rates (0.5 and 1.5 L s^−1^ kg^−1^_p_) and two different package designs. A comparison of the predicted fruit temperatures with the experimental data indicated that the developed simulator had a higher accuracy with *R* ^2^ and RMSE values in the ranges of .93866–.9986 and 0.07–0.62, respectively. Hence, it is possible to use this model for examining the precooling process in various conditions. The precooling process of apples was also studied inside a commercial package. Due to a long cooling time and considerable uniformity during the process, a new package was designed using the developed simulator. Based on the present results, the precooling process of apples was performed with a higher cooling rate in a shorter time (42%) with a lower cooling heterogeneity.

## Nomenclature

*A*: Pore area (m^2^)
C^+^: Universal constant for smooth walls
*C_µ_*, *C*_ɛ1_, *C*_ɛ2_, *σ_k_*, *σ_ɛ_*: Model constants
*C*_p_: Specific heat capacity (J kg^−1^ °C^−1^)
*D*: Hydraulic diameter (m)
*h*: Mesh element diameter
*k*: Thermal conductivity (W m^−1^ °C^−1^)
*k*_T_: Turbulent kinetic energy (m^2^/s^2^)
**n**: Outward normal to the surface
P: Pressure (Pa)
Pp: Pore perimeter (m)
Re: Reynolds number
RANS: Reynolds‐averaged Navier–Stokes
*T*: Temperature (K)
*t*: Time (s)
*U*: Velocity (m/s)
*u*: Average velocity (m/s)
*ρ*: Density (kg/m^3^)
*η*_T_: Turbulent viscosity
*η*: Dynamic viscosity (Pa.s)
*ε*: Dissipation rate of turbulence energy (m^2^/s^3^)

**Subscripts**
a: Air
p: Product

## CONFLICT OF INTEREST

The authors declare that they do not have any conflict of interest.

## ETHICAL APPROVAL

This study was approved by the University of Tabriz.

## INFORMED CONSENT

Written informed consent was obtained from all study participants.

## Supporting information

Fig S1

Fig S2
