# Supplementary material for: Exploration of heat and momentum transfer in turbulent mode during the precooling process of fruit
Source: Food Sci Nutr. 2020 Jul 2;8(8):4098–111. doi: 10.1002/fsn3.1682 (PMC7455990; doi:10.1002/fsn3.1682)
Supplement: Supplementary file 1 — Fig S1 [file FSN3-8-4098-s001.docx]

**Figure -1-suppinfo**: Mesh independence study
