# Supplementary figures and images for: Exploration of heat and momentum transfer in turbulent mode during the precooling process of fruit
Source: Food Sci Nutr. 2020 Jul 2;8(8):4098–111. doi: 10.1002/fsn3.1682 (PMC7455990; doi:10.1002/fsn3.1682)

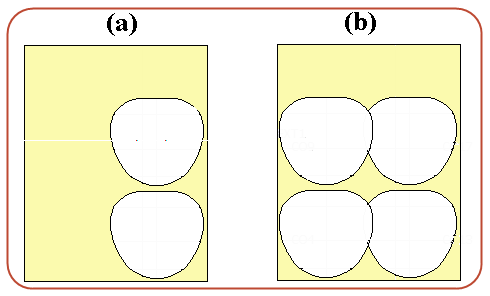


**Figure-2- Suppinfo:** Two cross section to calculate the Re number range

Supplement: Supplementary file 2 — Fig S2 [file FSN3-8-4098-s002.docx]
